# The New Kid on the Block: The Mechanisms of Action of Hyperleptinemia in Coronary Artery Disease and Atherosclerosis

**DOI:** 10.7759/cureus.15766

**Published:** 2021-06-20

**Authors:** Vernicia Hernandez, Kavaljeet Kaur, Mohamed W ElSharief, Sari W Al Hajaj, Ahmed M Ebrahim, Mirash Razack, David Dragas

**Affiliations:** 1 Internal Medicine, California Institute of Behavioral Neurosciences & Psychology, Fairfield, USA; 2 Pediatrics and Child Health, California Institute of Behavioral Neurosciences & Psychology, Fairfield, USA; 3 Surgery, California Institute of Behavioral Neurosciences & Psychology, Fairfield, USA; 4 Emergency Medicine, California Institute of Behavioral Neurosciences & Psychology, Fairfield, USA; 5 Internal Medicine, Wuhan University, Wuhan, CHN; 6 Internal Medicine, Al Ain Hospital, Al Ain, ARE; 7 Research, California Institute of Behavioral Neurosciences & Psychology, Fairfield, USA

**Keywords:** leptin, hypertension, atherosclerosis, coronary artery disease, myocardial infarction, obesity

## Abstract

Leptin is an adipocytokine that consists of 167 amino acids. It functions as a regulator of hunger and energy expenditure. Leptin loses its ability to carry out its physiological function at high serum levels, and many studies have associated this loss of function with the development of coronary artery disease (CAD). This literature review aims to outline the steps by which leptin leads to CAD and atherosclerosis. Two independent researchers extracted animal and human studies from PubMed and Google Scholar databases. We applied PubMed search builder options: pathology, pathophysiology, metabolism, and physiology to focus the search results. This study concluded that the mechanism by which leptin might lead to CAD via pressor and depressor effects on vascular tone, enhancing atherosclerotic plaques, and through numerous single nucleotide polymorphisms, the most common being that of the leptin receptor gene rs113701.

## Introduction and background

Coronary artery disease is a notable cause of death globally. There were 485.6 million coronary artery disease (CAD) cases in 2020; this represents an increase of 28.5% from 2017 [1]. Approximately 18 million lives are lost each year, accounting for 31% of deaths from all causes per annum [2]. According to the Heart Disease and Stroke Statistics - 2020 update, the economic burden of CAD in the United States has also increased. The United States observed a jump from 103.5 billion United States Dollars (USD) in 1996-1997 to 213.8 billion USD in 2014-2015 in the cost of managing patients with CAD [1]. The risk factors for CAD include cigarette smoking, sedentary lifestyle, metabolic syndrome, stress, depression, sleep disorders, obesity, diabetes mellites, and hypertension, with a body mass index of 30 or more being a leading cause [3].

The effects of obesity on the cardiovascular system

Obesity is associated with left ventricular hypertrophy, ventricular dysfunction, and diastolic heart failure [4]. Many scientists hypothesized that adipocytokines might be the link between obesity and CAD. Some examples of adipocytokines include leptin, interleukin 6 (IL-6), tumor necrosis factor (TNF), visfatin, adiponectin, resistin, and omentin [5]. This literature review addresses leptin.

Leptin's potential role in obesity-related cardiovascular disease

**Figure 1:**
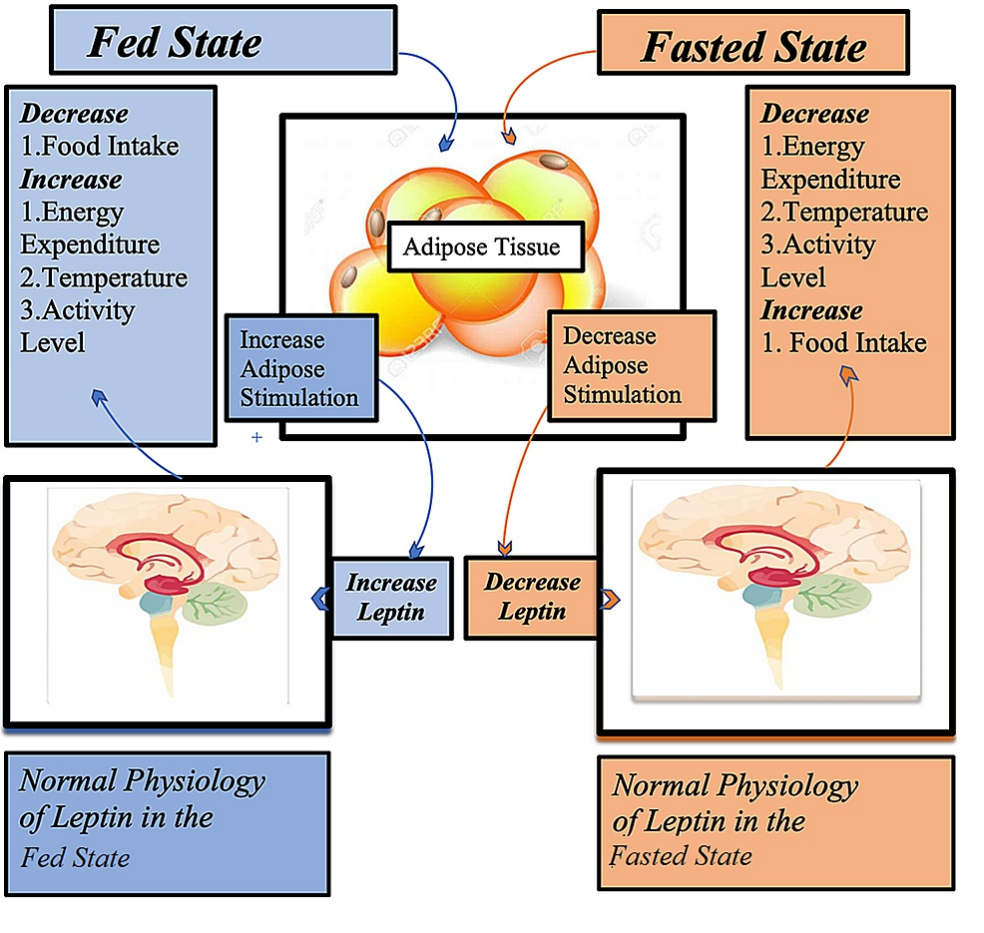
Showing the Physiological Actions of Leptin in the Fed and Fasted State

The leptin from white adipose tissue consists of 167 amino acids and is a product of the obese (ob) gene [6,7]. Leptin inhibits hunger and regulates energy expenditure through its interactions with the hypothalamus in the brain [8]. These actions decrease the volume of fat present in adipocytes [7]. Figure 1 below shows the physiological activities of leptin in the fed and fasted state.

A study on Indians showed that high leptin levels were associated with metabolic syndrome and increased CAD incidence [9]. Leptin is an essential determining factor in the occurrence, severity, degree, and complexity of lesions in patients with CAD [10]. Leptin is also implicated in myocardial infarction (MI) and stroke in men and women independently of conventional cardiovascular risk factors and BMI in the US population [11]. A study conducted by Chai et al. observed no statistically significant relationship between leptin and CAD; however, they concluded that high leptin levels in men should not be ignored [12].

Despite evidence of an association between hyperleptinemia and CAD, its pathophysiology is unclear. Appreciation of these concepts can result in new therapies for CAD. This literature review focuses on the mechanism of action of hyperleptinemia in CAD and atherosclerosis.

The data collected for this traditional review came from PubMed and Google Scholar databases. There was no set language restriction during the search process, but all articles selected had an English translation. This paper includes both human and animal studies. The researchers manually screened the reference lists of related original articles to identify more potential studies. We applied PubMed search builder options: pathology, pathophysiology, metabolism, and physiology to focus the search results. There were no additional inclusion or exclusion criteria used. The data extraction was performed independently by two researchers.

## Review

In the setting of hyperleptinemia, leptin loses its ability to execute its physiological functions. This concept is called the leptin resistance theory. The leptin resistance theory states that leptin's failure to prompt satiety is secondary to reduced hormone sensitivity [7]. Leptin resistance may contribute to CAD, hypertension, and atherosclerotic disease [13,14]. 

Leptin’s pressor effects on vascular tone

Leptin increases vascular tone by increasing sympathetic outflow. This pressor effect occurs with both intravenous and intracerebroventricular administration of leptin, as demonstrated in animal studies, with the intravenous route needing a one hundred times greater dose than the intracerebroventricular route to achieve the same degree of vasoconstriction [15]. Satoh et al. believed in leptin's centrally regulated pressor effects on vascular tone [16]. The surge of sympathetic activity observed occurs after leptin binds to leptin-receptors on the hypothalamus and result in an increase in heart rate, mean arterial blood pressure, and catecholamines, all of which disappeared when leptin infusion was stopped [17]. Additionally, short-term exposure to high levels of leptin proved to be inconsistent in elevating systematic blood pressure [18].

The Bielecka-Dabrowa et al. study showed that hypertension was associated with increased leptin and arterial stiffness. It concluded that leptin achieved its pressor effects through the sympathetic nervous systems [19]. Von Schnurbein et al. study states that the development of hypertension in obese patients was not dependent on leptin; still, leptin may have an additive effect on hypertension via increased sympathetic outflow [20].

Hyperleptinemia also has depressor effects on vascular tone. Rodrigues et al. observed phosphorylation of endothelial nitric oxide synthase (eNOS) and resulting nitric oxide (NO) production with leptin's infusion in rodents [21]. Matsuda et al. stated that leptin could cause arterial vasodilation in humans independent of NO [22]. In a study conducted by Kimura et al., researchers concluded that the NO-dependent pathway for leptin-induced vasorelaxation is dependent on the chloride ion channels [23]. Leptin also induced vascular NO production through the phosphoinositide 3-kinases (PI-3)-independent eNOS phosphorylation by the protein kinase B (Akt) pathway [24]. Leptin showed the capacity to upregulate eNOS phosphorylation and NO production through the adenosine monophosphate (AMP)-activated protein kinase (AMPK). Leptin's vasodilation properties contradict the belief that hyperleptinemia induces hypertension in obese patients [25].

The commonality seen in these studies is that the sympathetic nervous system is an essential mechanism by which Leptin has pressor effects on vascular tone. The results allude to a dose, length, and route-dependent leptin-induced vasoconstriction. These findings suggested that Leptin leads to hypertension, but the increased NO production and eNOS activity with depressor effects on vascular tone with leptin infusion contradict this theory. Based on these findings, this study concluded that leptin alone might have a neutral impact on vascular tone. However, the leptin resistance theory may account for hypertension seen in patients with hyperleptinemia, and therefore it should not be ignored.

Leptin’s role in atherosclerosis

Hyperleptinemia positively correlates to lower arterial compliance, acceleration of new blood vessel formation, osteoblastic differentiation, arterial wall calcification, and thrombosis generation [26,27]. Studies suggest that leptin expresses proinflammatory cytokine qualities by upregulating C-reactive protein (CRP) expression, cellular adhesion molecules, platelet tissue factor in human coronary endothelial cells, oxidative stress in human umbilical vein endothelial cells, inflammation, hypoxia, angiogenesis, and fibrosis in human perivascular adipose tissue [28-32]. Leptin also increases other inflammatory cells such as monocytes, leukocytes, and macrophages. These inflammatory cells produce many cytokines and inflammatory markers such as IL-6, TNFα, IL-12, and reactive oxygen species (ROS) [33-35]. These cytokines lead to atherosclerosis via endothelial dysfunction, which plays a vital role in CAD death and complications. CRP is an acute-phase protein that potentially enhances atherosclerotic plaques. CRP contributes to thrombosis development by increasing intercellular adhesion molecule-1 (ICAM-1), vascular cell adhesion molecule 1 (vCAM-1), E-selectin, monocyte chemoattractant protein-1 (MCP-1), endothelin 1 (ET-1), plasminogen activator inhibitor (PAI-1), and the proliferation and migration of smooth muscle cells, decreases tissue plasminogen activator (tPA) activity, and thrombomodulin expression [35]. Singh et al. demonstrated two crucial takeaway points in their study. They saw increased CRP concentrations at very high serum leptin levels and concluded; CRP did not predict cardiovascular disease independently, unlike leptin, which maintained a statistically significant predictive power. The increase CRP expression might be one of the underlying mechanisms behind atherosclerosis in hyperleptinemia [31].

**Figure 2:**
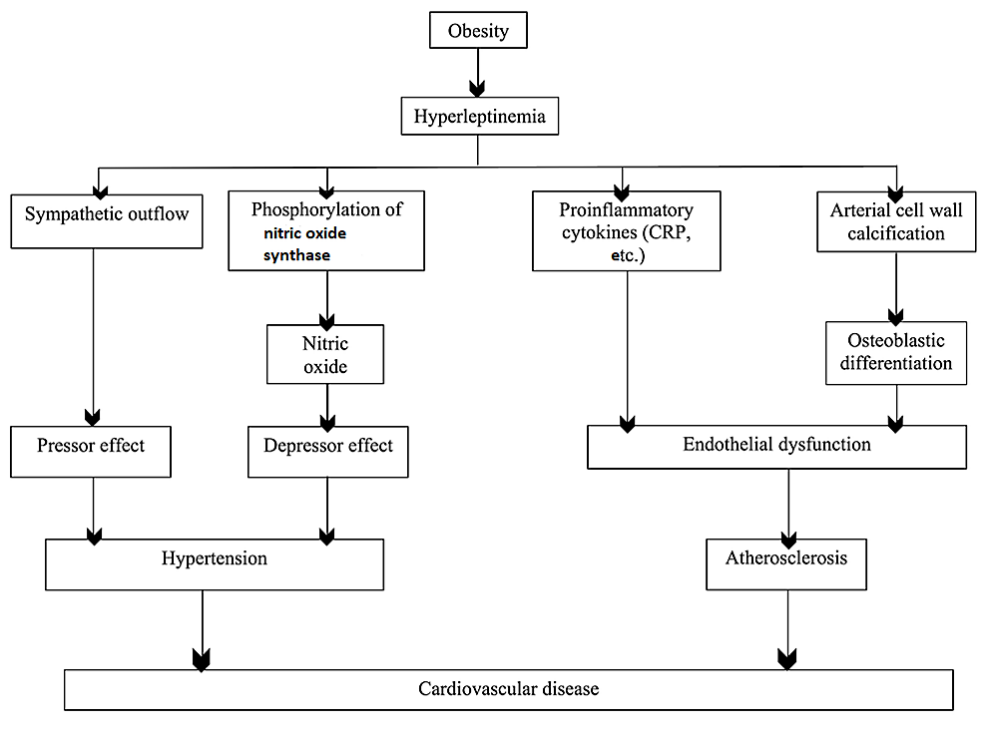
Effects of Hyperleptinemia on the Cardiovascular System CRP: C-reactive protein

Some studies suggest that leptin’s induction of atherosclerosis is receptor-dependent. Wild type and ob/ob mice showed that leptin's increment administration increased atherosclerosis and heart disease risk, independent of BMI, serum concentrations of lipid, and insulin sensitivity [36]. On the other hand, ob/ob deficient, leptin receptor-deficient, and apolipoprotein E (ApoE) mice demonstrated leptin's lack of protection against atherosclerosis [15,36]. These studies illustrate a link between ob/ob receptor, leptin, and atherosclerosis. Figure 2 below shows the effects of hyperleptinemia on the cardiovascular system.

Question for the future

How can the understanding of leptin’s effect on the cardiovascular system be applied to treatment, diagnosis, screening, and surveillance in individuals with and at high risk of CAD?

Single nucleotide polymorphisms of leptin and leptin receptor

Many studies report associations between leptin/leptin receptor interactions and CAD. Wang et al. conducted a study evaluating single nucleotide polymorphisms (SNPs) in leptin genes and leptin receptor (LEPR) genes [37]. The study aimed to uncover associations between different SNPs and CAD. The SNPs evaluated in the study included leptin genes (rs2167270 and rs7799039) and LEPR genes (rs6588147, rs1137100). A total of 384 North Chinese Han individuals were enrolled in the study, where 217 were patients diagnosed with CAD and 113 were healthy subjects. The results showed that: genetic variations at leptin rs7799039 and rs2167270 convey an increased risk of CAD in the northern Chinese population, and leptin rs2167270 and rs7799039 gene polymorphisms might affect predisposed individuals within the North Chinese population to CAD [37]. 

Nowzari et al. study assessed different SNPs of leptin, LEPR, and apelin receptor genes (APLNR) [38]. These included APLNR genes (rs11544374 and rs948847), a LEPR (rs1137101), and leptin (rs7799039) gene. The study subjects consisted of patients diagnosed with CAD and hypertension from the southeast Iranian population. The study concluded that all SNPs studied except APLNR rs11544374 were not associated with increased CAD or hypertension risk. Nowaria et al. deduced that APLNR rs11544374 gene polymorphism could represent a predisposition for CAD in the southeast Iranian population [38]. Khaki-Khatibi et al. studied demonstrated that Q223R (rs1137101) SNP of the LEPR gene conveys susceptibility to non-ST elevation myocardial infarction (NSTEMI). A total of 80 patients with confirmed STEMI (40 males and 40 females) and 80 healthy patients (40 males and 40 females) have participated in the study. The Khaki-Khatibi study concluded that Q223R (rs1137101) polymorphism could be a new prognostic indicator of NSTEMI [39]. 

An et al. sought to determine whether LEPR polymorphisms at Q223R (rs1137101) and K109R (rs1137100) were associated with increased risk of non-alcohol fatty liver disease (NAFLD) and coronary atherosclerosis in the Chinese Han population. A total of 554 patients diagnosed with non-alcohol fatty liver disease (NAFLD) (269 males, 285 females) and 421 patients diagnosed with coronary atherosclerosis (214 males, 207 females) participated in the study. An et al. selected all study subjects from the Qingdao area of China. The study concluded that LEPR Q223R (rs1137101) and A allele in the K109R (rs1137100) polymorphisms might confer a significant risk and an independent risk, respectively, for both NAFLD and CAD [40]. Table 1 below is a summary of the findings of the studies discussed under SNPs of leptin and leptin receptors.

**Table 1:** Showing Studies of Different Genetic Polymorphisms and Outcomes in Relation to the Cardiovascular System NAFLD: non-alcohol fatty liver disease; CAD: coronary artery disease; NSTEMI: non-ST elevation myocardial infarction; LEP: leptin N- SNPs were not assessed in the study. Y- SNPs were assessed in the study.

Author/Year	Number of Subjects/ Ethnicity	LEP Genes rs2167270	LEP Gene rs7799039	LEPR Genes rs6588147	LEPR Gene rs1137100	LEPR Gene rs1137101	Conclusion/Results
Wang et al. (2020) [37]	North Chinese 384	Y	Y	Y	Y	N	Genetic variations at LEP rs7799039 and rs2167270 increases risk and predispose individuals to CAD.
Nowzari et al. (2018) [38]	Iranian southeast 286	N	Y	N	N	Y	No association with increased risk of CAD and or hypertension
Khaki-Khatibi et al. (2018) [39]	Not Specified 160	N	N	N	N	Y	Rs1137101 polymorphism can be used as a new prognostic indicator of NSTEMI.
An et al. (2016) [40]	Chinese Han 975	N	N	N	Y	Y	Rs1137101 and Rs1137100 may confer a significant risk for both NAFLD and coronary atherosclerosis.
Saukko et al. (2010) [41]	Not Specified 526	N	N	N	Y	Y	Rs1137101 and Rs1137100 are associated with early-onset atherosclerosis and BMI systolic blood pressure, and intima-media thickness.

These studies show that genetic polymorphisms for the leptin gene and the LEPR gene may vary between races, geographical locations, and gender. Moreover, data gathered from individual studies may not apply to the worldwide population. In this study, LEPR gene rs1137101 was the most identified SNPs linked to CAD. Whether this is a practical approach to determining an individual's risk for CAD will depend on the availability of resources and overall cost.

Limitations

There are two limitations to this literature review: most of the studies included patients from the United States, Iran, and China. Whether the associations seen between leptin and different SNPs, atherosclerosis, and CAD could be applied to patients throughout the world requires more investigation. In addition, a degree of bias may have been introduced by excluding articles not published in a peer-reviewed journal and those manuscripts that are not available on electronic databases. Hence, our review should be viewed as a summary of the studies discussed and encourage additional research to evaluate the proposed findings.

## Conclusions

Leptin's role in CAD is poorly understood. Increased sympathetic outflow accounts for elevated blood pressure in obese patients, but leptin has mediated NO production, which contradicts this effect. Leptin binds to wild-type and ob/ob mice receptors, and its induction of inflammation and endothelial damage results in enhanced atherosclerotic plaque formation. Studies discussed in this review also showed associations between serum leptin concentration, hypertension, endothelial damage, atherosclerosis, inflammation, strokes, and CAD. Several SNPs in leptin genes and LEPR genes relay an increased risk for CAD, with LEPR gene rs1137101 being the most common SNP identified in this study. Many gaps exist in our understanding of leptin's pathophysiological pathways. Since cardiovascular disease carries a high economic, social, and financial burden, researchers should invest more time and money in understanding the biochemical mediators and receptors involved in its pathway to develop new targets for drugs used in the treatment of CAD.
